# Perspektive Prävention: Psychische Gesundheit von Schülerinnen und Schülern in Deutschland

**DOI:** 10.1007/s00103-023-03674-8

**Published:** 2023-03-10

**Authors:** Franziska Reiß, Ann-Kathrin Napp, Michael Erhart, Janine Devine, Kevin Dadaczynski, Anne Kaman, Ulrike Ravens-Sieberer

**Affiliations:** 1 Zentrum für Psychosoziale Medizin, Klinik für Kinder- und Jugendpsychiatrie, -psychotherapie und -psychosomatik, Universitätsklinikum Hamburg-Eppendorf, Hamburg, Deutschland; 2 Alice Salomon Hochschule, Berlin, Deutschland; 3 Apollon Hochschule der Gesundheitswirtschaft, Bremen, Deutschland; 4 Fachbereich Gesundheitswissenschaften, Hochschule Fulda, Fulda, Hessen Deutschland; 5 Zentrum für Psychosoziale Medizin, Klinik für Kinder- und Jugendpsychiatrie, -psychotherapie und -psychosomatik, Forschungssektion Child Public Health, Universitätsklinikum Hamburg-Eppendorf, Martinistr. 5, 20246 Hamburg, Deutschland

**Keywords:** Kinder und Jugendliche, COPSY-Studie, Wohlbefinden, Gesundheitsförderung, COVID-19-Pandemie, Children and adolescents, COPSY-Study, Well-being, Health promotion, COVID-19 pandemic

## Abstract

**Hintergrund:**

Die COVID-19-Pandemie hat das Lernen und die Gesundheit von Kindern und Jugendlichen beeinflusst. Ziel des Beitrags ist, psychische Auffälligkeiten von SchülerInnen im Pandemieverlauf, familiäre Belastungen sowie Unterstützungsbedarf in Abhängigkeit von der Schulform zu untersuchen. Ansätze schulischer Prävention und Gesundheitsförderung werden diskutiert.

**Methodik:**

Datengrundlage sind die bevölkerungsbezogene COPSY(COrona und PSYche)-Studie (T1: 05/2020 – T4: 02/2022) und die BELLA-Studie (T0, präpandemischer Vergleich). Je Messzeitpunkt (T) wurden etwa 1600 Familien mit Schulkindern im Alter von 7 bis 19 Jahren befragt. Psychische Auffälligkeiten wurden mittels SDQ erfasst, familiäre Belastungen und Unterstützungsbedarfe mittels Einzelitems im Elternbericht.

**Ergebnisse:**

Psychische Auffälligkeiten haben bei SchülerInnen aller Schulformen zugenommen und stabilisieren sich seither auf hohem Niveau. Besonders betroffen sind GrundschülerInnen (Anstieg von 16,9 % präpandemisch auf 40,0 % zu T2), v. a. bei Verhaltensauffälligkeiten (11,7 % auf 24,6 %) und Hyperaktivität (13,9 % auf 34,0 %). SchülerInnen der Haupt‑/Real‑/Gesamtschule zeigen ebenfalls verstärkte psychische Auffälligkeiten (21,4 % auf 30,4 %). Pandemiebedingte Belastungen sind durchgehend hoch, ebenso der Unterstützungsbedarf von Familien, der sich v. a. an Schule/Lehrende sowie ExpertInnen richtet.

**Diskussion:**

Es besteht ein hoher Bedarf an Maßnahmen der psychischen Gesundheitsförderung und Prävention im Setting Schule. Diese sollten ab dem Grundschulalter im Sinne eines Whole School Approach auf verschiedenen Ebenen ansetzen und außerschulische Akteure einbeziehen. Es bedarf verbindlicher gesetzlicher Vorgaben in allen Bundesländern, um Rahmbedingungen und Strukturen der schulischen Gesundheitsförderung und Prävention, einschließlich der dafür benötigten Ressourcen, zu schaffen.

**Zusatzmaterial online:**

Zusätzliche Informationen sind in der Online-Version dieses Artikels (10.1007/s00103-023-03674-8) enthalten.

## Abstract (en)

**Background:**

The COVID-19 pandemic has impacted the learning and health of children and adolescents. The aim of this paper is to examine school students’ mental health problems, family burden, and support needs during the pandemic depending on the type of school. Approaches to school-based prevention and health promotion are discussed.

**Methods:**

Findings are based on data from the population-based COPSY study (T1: 05/2020 – T4: 02/2022) and the BELLA study (T0, pre-pandemic comparison). Approximately 1600 families with children aged 7 to 19 years were surveyed at each measurement point (T). Mental health problems were assessed using the SDQ, while family burden and support needs were captured with individual items in the parent report.

**Results:**

Mental health problems increased among students in all school types at the beginning of the pandemic and have stabilized at a high level. Elementary school students are particularly affected (increase from 16.9% pre-pandemic to 40.0% at T2), especially in behavioral problems (11.7% to 24.6%) and hyperactivity (13.9% to 34.0%). Secondary school students also show higher levels of mental health problems (21.4% to 30.4%). Pandemic-related burden is consistently high, as is the need for family support received from schools/teachers and experts.

**Discussion:**

There is a high need for mental health promotion and prevention measures in the school setting. These should start at primary school age in the sense of a “whole school approach” at different levels and include external stakeholders. In addition, binding legal requirements are needed in all federal states to create framework conditions and structures for school-based health promotion and prevention, including access to necessary resources.

## Einleitung

Kinder und Jugendliche stellen mit Blick auf ihre Gesundheit und ihr Gesundheitsverhalten eine vulnerable Gruppe dar, die besonders in Krisenzeiten besonderer Aufmerksamkeit bedarf, da sie sich nicht selbst schützen kann. Bereits vor Beginn der COVID-19-Pandemie zeigten etwa 18 % der Kinder und Jugendlichen in Deutschland Hinweise auf psychische Auffälligkeiten [1, 2]. Studien belegen einen Anstieg von pandemiebedingten Belastungen und Risikofaktoren, was wiederum zu einer Zunahme von psychischen Auffälligkeiten im Kindes- und Jugendalter geführt hat [3–5]. Eine Studie aus Nordrhein-Westfalen hat gezeigt, dass sich das Belastungserleben und die psychische Gesundheit in der Pandemie zwischen den Schulformen unterscheiden [6]. Bundesweite Ergebnisse liegen derzeit nicht vor.

Zu den häufigsten psychischen Erkrankungen bei SchülerInnen zählen Angststörungen, Depressionen, Störungen des Sozialverhaltens sowie Lernstörungen [7]. Die im Kindes- und Jugendalter erstmals auftretenden psychischen Erkrankungen bergen zudem das Risiko, bis ins Erwachsenenalter zu persistieren bzw. zu exazerbieren [8]. Zudem erhöhen psychische Störungen nicht nur das Risiko für eine Klassenwiederholung, sondern auch für Schulabsentismus und -abbruch [7]. Daher sind aus gesellschaftlicher und gesundheitsökonomischer Sicht die Gesundheitsförderung und Prävention im Kindes- und Jugendalter besonders relevant, um die Gesundheit, Bildungschancen und damit auch das Arbeitseinkommen der zukünftigen Erwachsenengeneration zu fördern und das Risiko psychischer Erkrankungen zu reduzieren. Umfassende Forschungsarbeiten haben personale, soziale, familiäre und umweltbezogene Risikofaktoren und Ressourcen identifiziert, die für ein gesundes Aufwachsen von Kindern und Jugendlichen entscheidend sind [9–13]. Die Familie, die Beziehung zu Gleichaltrigen und das schulische Umfeld sind hierbei von besonderer Bedeutung.

Die psychische Gesundheit von Heranwachsenden hat daher eine hohe Public-Health-Relevanz. Dabei ist die Schule seit Verabschiedung der Ottawa-Charta im Jahr 1986 weltweit zu einem zentralen Setting der Gesundheitsförderung und Prävention geworden [14]. Die schulische Gesundheitsförderung ist nicht nur Bestandteil der Praxis (Schule), sondern auch der Forschung (Evidenz) und Politik (Gesetzgebung) [15]. Sie umfasst eine Vielzahl von Ansätzen, die sich sowohl auf die Präventionsebenen (Verhalten, Verhältnisse), Zielgruppen (SchülerInnen, Lehrkräfte, Eltern) und Themen (z. B. körperliche Aktivität, psychische Gesundheit, Ernährung) beziehen [16]. Schule wird dabei nicht nur als ein Ort des Lernens verstanden, sondern dient auch der Sensibilisierung für die Themen psychische und körperliche Gesundheit sowie gesundheitsförderliches Verhalten [17].

In Bezug auf Maßnahmen der Prävention und Gesundheitsförderung kommt der Schule gerade in Krisenzeiten eine besondere Bedeutung zu. Konzepte wie die „gesundheitsfördernde Schule“ werden beispielsweise durch die Weltgesundheitsorganisation (WHO) oder das Schools-for-Health-in-Europe(SHE)-Netzwerk favorisiert [18, 19]. Sie setzen auf einen ganzheitlichen Ansatz (Whole School Approach), um die soziale und emotionale Entwicklung von SchülerInnen zu fördern. Trotz unterschiedlicher konzeptueller Akzentuierungen setzen ganzheitliche Ansätze an mindestens 3 Ebenen an: 1) Lehren, Lernen und Curriculum, 2) Schulkultur und schulische Umwelt sowie 3) Einbezug außerschulischer Dienste und Kooperationspartner (einschließlich der Familie) [20].

In einer Metaanalyse zu Evaluationsstudien von 30 verschiedenen Interventionen im Rahmen des Whole School Approach konnten Goldberg et al. [21] signifikante, wenn auch geringe Verbesserungen in der sozialen, emotionalen und verhaltensbezogenen Entwicklung sowie bei internalisierenden Symptomen von SchülerInnen feststellen. In einer etwas älteren Übersichtarbeit konnte gezeigt werden, dass wirksame Interventionen verschiedene Merkmale aufweisen. Dazu zählen u. a. pädagogische Vermittlungsfähigkeiten, die Ausrichtung an einem positiven Verständnis von psychischer Gesundheit, Fortbildung für Lehrende und Eltern, verschiedene Interventionsformen, ein lang angelegter Interventionszeitraum oder auch die Ausrichtung an einem ganzheitlichen Ansatz [22]. Ein kontinuierliches Gesundheitsmonitoring zur psychischen Gesundheit der Kinder und Jugendlichen sowie deren Bedarfe sollte hierbei grundlegend sein.

Der vorliegende Beitrag hat das Ziel, einen Überblick über psychische Auffälligkeiten von SchülerInnen im Verlauf der COVID-19-Pandemie, pandemiebedingte familiäre Belastungen und den familiären Unterstützungsbedarf zu geben. Die zentralen Fragestellungen des Beitrags lauten:

1. Wie hat sich die psychische Gesundheit von SchülerInnen verschiedener Schulformen in Deutschland seit dem Beginn der COVID-19-Pandemie verändert?
2. Wie belastet fühlen sich Familien durch die Veränderungen im Zusammenhang mit der COVID-19-Pandemie?
3. Welchen Unterstützungsbedarf sehen Eltern und welche Angebote zur Prävention und Gesundheitsförderung an Schulen gibt es?

In diesem Zusammenhang werden Ansätze der schulischen Prävention und Gesundheitsförderung vorgestellt sowie weiterführende Implikationen für die Prävention psychischer Auffälligkeiten von Heranwachsenden aufgezeigt.

## Methoden

### Studiendesign und Stichprobe

Die Datengrundlage bildet die bundesweite bevölkerungsbezogene COPSY-Längsschnittstudie (COrona und PSYche). Im Zeitraum von Mai 2020 bis Februar 2022 wurden zu 4 Messzeitpunkten Familien von SchülerInnen im Alter von 7 bis 19 Jahren zu ihrer psychischen Gesundheit und ihrem Gesundheitsverhalten befragt. Vergleichsdaten zu der Zeit vor der Pandemie bietet die bundesweite bevölkerungsbezogene BELLA-Studie (BEfragung zum seeLischen WohLbefinden und VerhAlten; [23]). Die BELLA-Studie ist das Modul zur psychischen Gesundheit der Studie zur Gesundheit von Kindern und Jugendlichen in Deutschland (KiGGS), welche seit 2003 am Robert Koch-Institut durchgeführt wird. Die COPSY-Studie ist als prospektive Längsschnittstudie konzipiert, d. h., es werden sowohl Teilnehmende wiederholt befragt (Längsschnitt) als auch zu jedem Befragungszeitpunkt neue Teilnehmende hinzugewonnen (repräsentativer Querschnitt), um Ausfälle durch ausscheidende Teilnehmende zu kompensieren und die Repräsentativität zu erhalten. Die Stichproben der COPSY-Studie wurden gewichtet, um in den wesentlichen Merkmalen der Bevölkerungsstruktur von Familien mit 7‑ bis 17-jährigen Kindern in Deutschland gemäß Mikrozensus 2018 zu entsprechen.

In diesem Beitrag wurden nur Angaben von Eltern mit Kindern, die zum Zeitpunkt der Befragung eine Schule besuchten, ausgewertet. Für den Vergleich zu der Zeit vor der Pandemie wurden Daten der BELLA -Studie aus dem Erhebungszeitraum 2014 bis 2017 verwendet. Weitere Informationen zu Design, Methodik und Ergebnissen der BELLA-Studie finden sich in Otto et al. [23].

### Instrumente

Soziodemografische Angaben zu Geschlecht und Alter der befragten Elternteile sowie ihrer Kinder wurden im Elternbericht erfasst. Zudem wurde der Migrationshintergrund erfragt. Das elterliche Bildungsniveau wurde mittels der international etablierten CASMIN-Klassifikation (Comparative Analysis of Social Mobility in Industrial Nations) in niedrig, mittel und hoch eingeteilt [24].

Die Schulform und Klassenstufe der Kinder wurden mittels einzelner Items erfragt. Eine Kategorisierung der Schulform erfolgte in (1) *Grundschule*, (2) *Schule mit Haupt- und/oder Realschulbildungsgang, Gesamtschule,* (3) *Gymnasium oder Fachoberschule* sowie (4) *Förder- oder Sonderschule.* Eltern, die zuvor angegeben hatten, dass auf ihr Kind eine andere Schulform zutreffe, wurden gebeten diese zu benennen. Die Freitextangabe wurde entsprechend der Klassenstufe manuell einer der 4 Kategorien zugeordnet. Dies betraf 1,65 % (*n* = 25) in der ersten, 1,80 % (*n* = 27) in der zweiten, 1,89 % (*n* = 26) in der dritten und 2,41 % (*n* = 35) der Angaben in der vierten COPSY-Befragung.

Psychische Auffälligkeiten wurden mittels 4 Subskalen und der Gesamtskala des etablierten Strenghts and Difficulties Questionnaire (SDQ) im Elternbericht erfasst. Die 4 Subskalen des SDQ erfassen Verhaltensprobleme, emotionale Probleme, Hyperaktivität und Probleme mit Gleichaltrigen [25]. Eine Kategorisierung der Kinder- und Jugendlichen in *unauffällig* und *auffällig bzw. grenzwertig* erfolgte anhand der deutschen Cut-off-Werte [26].

Eingesetzt wurden darüber hinaus selbst entwickelte Items zum Belastungserleben der Eltern in der Pandemie („Wie belastend waren Veränderungen im Zusammenhang mit der Corona-Pandemie für Sie insgesamt?“, mit 5‑stufiger Antwortskala von 1 = *gar nicht belastend* bis 5 = *äußerst belastend*) sowie zum Unterstützungsbedarf der Eltern im Umgang mit ihrem Kind („Würden Sie sich im Umgang mit Ihrem Kind während der Corona-Pandemie Unterstützung wünschen?“, mit 4‑stufiger Antwortskala von 1 = *nie* bis 4 = *Ja, immer*). Eltern, die angaben, sich zumindest manchmal Unterstützung zu wünschen, wurden zudem nach der Art der Unterstützung gefragt („Wie möchten Sie diese Unterstützung bekommen?“, 12 Antwortoptionen, z. B. durch Lehrende/Schule, Bekannte/Familie, Podcasts, persönliche Unterstützung von ExpertInnen, schriftliches Onlinematerial).

### Statistische Analysen

Die Auswertung erfolgte mittels deskriptiver Statistiken (absolute und relative Häufigkeiten, Mittelwerte und Standardabweichungen). Außerdem wurden bivariate Tests (Chi-Quadrat-Tests) zum Vergleich der kategorisierten Schulformen durchgeführt. Als Effektgrößenmaß für die Unterschiede wurde Cramers V herangezogen, wobei gemäß Cohen V = 0,1/0,3/0,5 einem kleinen/mittleren/starken Effekt entspricht [27]. Signifikante Gruppenunterschiede wurden bei einem Signifikanzniveau von *p* < 0,05 angenommen. Alle Analysen erfolgten mit SPSS Version 27 (IBM, Armonk, NY, USA).

## Ergebnisse

### Soziodemografie

An der ersten COPSY-Befragung (Mai/Juni 2020) nahmen *n* = 1517 (95,65 % von *N* = 1586) Familien mit Schulkindern teil, an der zweiten Befragung (Dezember 2020–Januar 2021) nahmen *n* = 1497 (92,12 % von *N* = 1625) Familien teil. Zu diesen Zeiten galt in Deutschland ein bundesweiter Lockdown. An der dritten Befragung (September/Oktober 2021) nahmen *n* = 1377 (85,11 % von *N* = 1618) und an der vierten Befragung (Februar 2022) nahmen *n* = 1454 (87,15 % von *N* = 1668) Familien mit Schulkindern teil. Die teilnehmenden SchülerInnen der 4 COPSY-Befragungen waren im Mittel zwischen 12 und 13 Jahre alt und zu gleichen Teilen männlich und weiblich. Das durchschnittliche Alter der Eltern betrug 44 Jahre und zu allen Befragungszeitpunkten nahmen mehr weibliche als männliche Elternteile teil (54,3–58,6 %). Die Mehrheit der befragten Eltern hatte keinen Migrationshintergrund (81,1–84,0 %) und ein mittleres Bildungsniveau (56,5–58,0 %).

Präpandemische Vergleichsdaten der BELLA-Studie umfassen *n* = 1483 (93,94 % von *N* = 1580) teilnehmende Familien mit Kindern im Alter von 7 bis 19 Jahren, die zum Zeitpunkt der Befragung eine Schule besuchten. Weitere soziodemografische Angaben finden sich in Tab. 1.

**Table 1:** 

	*Welle 1*^*a*^ *n* *=* *1517*	*Welle 2*^*a*^ *n* *=* *1497*	*Welle 3*^*a*^ *n* *=* *1377*	*Welle 4*^*a*^ *n* *=* *1454*
	*n (%)*	*n (%)*	*n (%)*	*n (%)*
**Alter des Kindes in Jahren (M, SD)**	12,05 (3,23)	12,35 (3,16)	12,58 (3,05)	12,25 (3,42)
*Geschlecht des Kindes*				
Männlich	753 (49,7 %)	757 (50,6 %)	661 (48, 1 %)	715 (49,2 %)
Weiblich	762 (50,3 %)	738 (49,3 %)	704 (51,2 %)	729 (50,2 %)
Divers	1 (0,1 %)	1 (0,1 %)	9 (0,7 %)	9 (0,6 %)
**Alter der Eltern in Jahren (M, SD)**	*43,84 (7,37)*	*43,77 (7,37)*	*43,89 (7,47)*	*43,85 (7,63)*
*Geschlecht der Eltern*				
Männlich	692 (45,6 %)	672 (44,9 %)	567 (41,2 %)	643 (44,2 %)
Weiblich	824 (54,3 %)	823 (55,0 %)	807 (58,6 %)	809 (55,6 %)
Divers	1 (0,1 %)	2 (0,1 %)	3 (0,2 %)	2 (0,1 %)
*Migrationshintergrund*				
Nein	1270 (83,7 %)	1242 (84,0 %)	1106 (81,1 %)	1182 (82,1 %)
Ja	247 (16,3 %)	237 (16,0 %)	257 (18,9 %)	258 (17,9 %)
*Bildung*				
Untere Bildungskategorie	268 (18,0 %)	263 (17,9 %)	243 (18,0 %)	230 (16,1 %)
Mittlere Bildungskategorie	841 (56,6 %)	829 (56,5 %)	783 (58,0 %)	815 (57,0 %)
Obere Bildungskategorie	378 (25,4 %)	374 (25,0 %)	325 (24,1 %)	384 (26,9 %)
Keine Information	30 (2,0 %)	31 (2,1 %)	26 (1,9 %)	25 (1,7 %)
*Schulform*				
Grundschule	537 (35,4 %)	453 (30,3 %)	331 (24,0 %)	497 (34,2 %)
Schule mit Haupt- und/oder Realschulbildungsgang, Gesamtschule	540 (35,6 %)	560 (37,4 %)	524 (38,1 %)	450 (30,9 %)
Gymnasium oder Fachoberschule	390 (25,7 %)	432 (28,9 %)	464 (33,7 %)	445 (30,6 %)
Förderschule oder Sonderschule	50 (3,3 %)	52 (3,5 %)	58 (4,2 %)	62 (4,3 %)

*M* Mittelwert, *SD* Standardabweichung

^a^ Erhebungszeiträume der COPSY-Studie: Welle 1 (Mai–Jun. 2020), Welle 2 (Dez. 2020–Jan. 2021), Welle 3 (Sep.–Okt. 2021), Welle 4 (Feb. 2022)

### Psychische Auffälligkeiten

**Figure 1:**
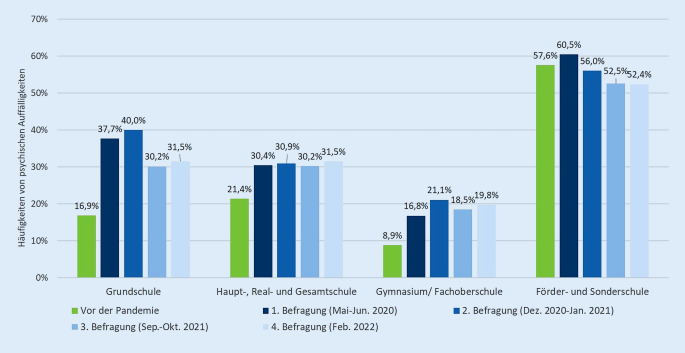

Psychische Auffälligkeiten nahmen im Vergleich zur Zeit vor der Pandemie bei SchülerInnen aller Schulformen zu (Abb. 1). Ein deutlicher Anstieg der psychischen Auffälligkeiten zeigte sich bei GrundschülerInnen von 16,9 % vor der Pandemie auf bis zu 40,0 % im zweiten Lockdown im Winter 2020/2021 sowie bei SchülerInnen der Gesamt‑, Haupt- und Realschulen von 21,4 % vor der Pandemie auf bis zu 30,9 % im zweiten Lockdown im Winter 2020/2021.

SchülerInnen an Gymnasien und Fachoberschulen waren im Pandemieverlauf mehr als doppelt so häufig von psychischen Auffälligkeiten betroffen wie vor der Pandemie (8,9 % vor der Pandemie vs. 21,1 % 2. Befragung). Im Pandemieverlauf unterschieden sich die Schulformen zu allen Befragungszeitpunkten signifikant (1. Befragung: Cramers V = 0,208, *p* < 0,001; 2. Befragung: Cramers V = 0,180, *p* < 0,001; 3. Befragung: Cramers V = 0,165, *p* < 0,001; 4. Befragung: Cramers V = 0,159, *p* < 0,001).

Am häufigsten traten psychische Auffälligkeiten bei FörderschülerInnen auf. Die höchste Prävalenz von 60,5 % wurde in der ersten COPSY-Befragung zu Beginn der Pandemie berichtet, in der dritten und vierten COPSY-Befragung (Herbst/Winter 2021 und Februar 2022) lag sie jedoch unter dem präpandemischen Niveau. SchülerInnen, die das Gymnasium oder eine Fachoberschule besuchten, zeigten zu allen Befragungszeitpunkten die vergleichsweise geringsten psychischen Auffälligkeiten. Während die Prävalenz in Gesamt‑, Haupt- und Realschulen konstant bei ca. 30 % lag, war sie neben den Förderschulen auch in den Grundschulen leicht rückläufig. Sie hat sich hier jedoch im Verlauf auf einem 10–15 % höheren Niveau im Vergleich zur Zeit vor der COVID-19-Pandemie stabilisiert.

Bei den einzelnen Subskalen psychischer Auffälligkeiten fanden sich ebenfalls Unterschiede im Vergleich zur Zeit vor der Pandemie. Bei GrundschülerInnen traten in der Pandemie vermehrt Verhaltensprobleme (11,7 % vs. 26,1 % 2. Befragung) und Hyperaktivität (13,9 % vs. 34,0 % 1. Befragung) auf. Im Vergleich zur Zeit vor der Pandemie nahmen Probleme im Umgang mit Gleichaltrigen vor allem bei Haupt‑, Real- und GesamtschülerInnen zu (11,5 % vs. 29,5 % 2. Befragung), aber auch bei SchülerInnen der Gymnasien konnte ein Anstieg verzeichnet werden (9,1 % vs. 25,4 % 2. Befragung). Emotionale Probleme fanden sich bei knapp einem Drittel der Förder- und SonderschülerInnen und damit doppelt so häufig wie vor der Pandemie (15,2 %). Auch bei SchülerInnen der Grundschule und am Gymnasium zeigte sich ein Anstieg emotionaler Probleme. Während diese bei den GrundschülerInnen ab der 3. COPSY-Befragung wieder rückläufig waren, verzeichneten die anderen Schulformen einen erneuten Anstieg.

Verhaltensprobleme im Umgang mit Gleichaltrigen (43,8 %) sowie Hyperaktivität (53,1 %) traten bereits vor der Pandemie bei den Förder- und SonderschülerInnen deutlich häufiger auf. In der Pandemie nahmen diese jedoch um mehr als 10 Prozentpunkte ab (28,6 % Probleme mit Gleichaltrigen in der 1. Befragung; 39,0 % Hyperaktivität in 3. Befragung). Alle anderen Schulformen verzeichneten teils deutliche Zunahmen bei Unaufmerksamkeit und Hyperaktivität. Tab. 2 zeigt die Prävalenzen der verschiedenen Subskalen des SDQ für alle COPSY-Befragungen. Eine grafische Darstellung findet sich im zusätzlichen Onlinematerial (Abbildung Z1).

**Table 2:** 

	BELLA^a^ (%)	COPSY Welle 1^a^ (%)	COPSY Welle 2^a^ (%)	COPSY Welle 3^a^ (%)	COPSY Welle 4^a^ (%)
*Verhaltensprobleme*					
Grundschule	11,7	24,6	26,1	21,6	21,6
Schule mit Haupt- und/oder Realschulbildungsgang, Gesamtschule	16,2	19,1	17,8	16,9	16,7
Gymnasium oder Fachoberschule	9,4	10,8	13,6	12,9	8,9
Förderschule oder Sonderschule	27,3	35,7	34,0	24,1	31,7
Signifikanzniveau (*p*)	*0,001*	*<* *0,001*	*<* *0,001*	*0,01*	*<* *0,001*
Effektstärke (Cramers V)	*0,06*	*0,152*	*0,139*	*0,091*	*0,159*
*Emotionale Probleme*					
Grundschule	16,2	26,7	29,5	25,3	25,3
Schule mit Haupt- und/oder Realschulbildungsgang, Gesamtschule	20,6	19,7	22,6	26,8	26,8
Gymnasium oder Fachoberschule	11,5	13,9	19,6	20,8	21,4
Förderschule oder Sonderschule	15,2	31,0	30,0	33,9	34,4
Signifikanzniveau (*p*)	*0,005*	*<* *0,001*	*0,004*	0,066	0,081
Effektstärke (Cramers V)	*0,05*	*0,130*	*0,094*	0,072	0,068
*Probleme mit Gleichaltrigen*					
Grundschule	9,5	18,4	23,7	18,7	17,1
Schule mit Haupt- und/oder Realschulbildungsgang, Gesamtschule	11,5	24,8	29,5	26,0	27,6
Gymnasium oder Fachoberschule	9,1	20,4	25,4	22,1	22,8
Förderschule oder Sonderschule	43,8	28,6	44,0	32,8	34,9
Signifikanzniveau (*p*)	*0,01*	*0,045*	*0,007*	*0,025*	*<* *0,001*
Effektstärke (Cramers V)	*0,13*	*0,073*	*0,090*	*0,082*	*0,117*
*Unaufmerksamkeit/Hyperaktivität*					
Grundschule	13,9	34,0	29,5	23,8	27,6
Schule mit Haupt- und/oder Realschulbildungsgang, Gesamtschule	14,8	20,4	20,6	18,5	16,7
Gymnasium oder Fachoberschule	3,7	12,0	9,3	9,3	8,9
Förderschule oder Sonderschule	53,1	50,0	46,0	39,0	42,2
Signifikanzniveau (*p*)	*0,01*	*<* *0,001*	*<* *0,001*	*<* *0,001*	*<* *0,001*
Effektstärke (Cramers V)	*0,09*	*0,229*	*0,217*	*0,181*	*0,225*

^a^ Erhebungszeiträume der BELLA-Studie (2014–2017) und der COPSY-Studie: Welle 1 (Mai–Jun. 2020), Welle 2 (Dez. 2020–Jan. 2021), Welle 3 (Sep.–Okt. 2021), Welle 4 (Feb. 2022); *p* = Phi-Koeffizient

### Belastungserleben

Zu allen 4 Erhebungen fühlte sich der Großteil der befragten Eltern durch die Veränderungen im Zusammengang mit der COVID-19-Pandemie belastet. Im Vergleich der Schulformen waren Eltern mit Kindern in der Grundschule (86,6–87,7 %) und in der Förder- bzw. Sonderschule (84,0–88,9 %) am stärksten belastet. Signifikante Unterschiede zwischen den Schulformen fanden sich jedoch nur in der ersten und in der vierten COPSY-Befragung (1. Befragung: Cramers V = 0,151, *p* < 0,001; 4. Befragung: Cramers V = 0,102; *p* = 0,002).

### Unterstützungsbedarf

**Figure 2:**
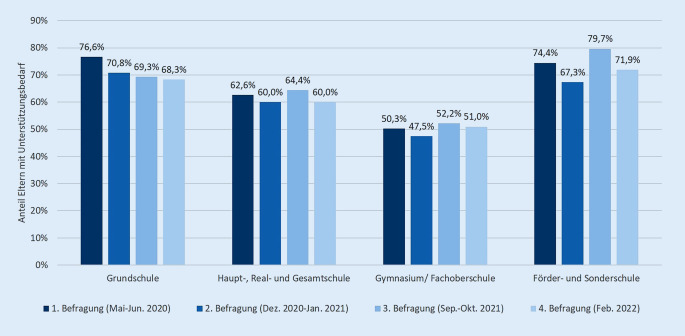

Ein Großteil der Eltern hatte 2020 bis 2022 einen Unterstützungsbedarf im Umgang mit ihren Kindern in der Pandemie. Es fanden sich deutliche Unterschiede zwischen den Schulformen (Abb. 2). Insbesondere Eltern von Sonder- oder FörderschülerInnen (79,7 % zur 3. Befragung) sowie GrundschülerInnen (76,6 % zur 1. Befragung) wünschten sich Unterstützung. Etwa die Hälfte der Eltern von GymnasialschülerInnen gab einen Unterstützungsbedarf an (52,2 % zur 3. Befragung).

**Figure 3:**
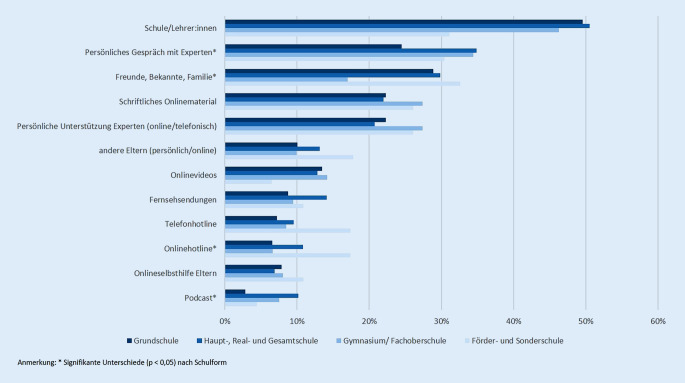

Gaben Eltern einen Unterstützungsbedarf an, wünschten sie sich vor allem Unterstützung beim Umgang mit schulischen Anforderungen ihres Kindes (53,3–80,7 %) sowie den Gefühlen (17,2–60,9 %) und dem Verhalten des Kindes (14,7–56,5 %). Hier zeigten sich deutliche Unterschiede zwischen den Schulformen, wobei Eltern von Kindern auf dem Gymnasium jeweils den geringsten und solche mit Kindern auf Sonder- oder Förderschulen den höchsten Unterstützungsbedarf aufwiesen. Zu Beginn der Pandemie wünschte sich zudem rund ein Drittel der Eltern Unterstützung bei der Rückkehr des Kindes aus der Isolation (25,8–38,8 %). Bei der Art der Unterstützung wünschten sich Eltern über alle Schulformen hinweg vor allem Unterstützung durch die Schule bzw. den Kontakt zu LehrerInnen (31,1–50,5 %; Abb. 3). Auch persönliche Gespräche mit ExpertInnen (24,5–34,9 %) sowie Unterstützung durch FreundInnen, Bekannte und andere Familienmitglieder wurden häufig angegeben (17,0–32,6 %). Onlineangebote, die schriftliche Form oder der Austausch mit ExpertInnen wurden jeweils von einem Viertel der Eltern ausgewählt. Der Bedarf an weiteren Unterstützungsarten ist in Abb. 3 dargestellt. Signifikante Unterschiede zwischen den Schulformen finden sich nur in Bezug auf das persönliche Gespräch mit ExpertInnen (Cramers V = 0,105, *p* = 0,021), Podcasts (Cramers V = 0,128, *p* = 0,002), Onlinehotlines (Cramers V = 0,102; *p* = 0,027) sowie den Austausch mit FreundInnen, Bekannten und Familie (Cramers V = 0,123, *p* = 0,004).

## Diskussion

Die Ergebnisse der COPSY-Studie zeigen, dass mit dem Beginn der COVID-19-Pandemie psychische Auffälligkeiten bei SchülerInnen deutlich zugenommen haben und sich seither auf einem hohen Niveau stabilisieren. Um mögliche daraus folgende psychische Erkrankungen dieser Kinder und Jugendlichen in den nächsten Jahren zu vermeiden resp. ihre psychische Gesundheit zu stärken, werden Maßnahmen der schulischen Gesundheitsförderung und Prävention mit zunehmender Dauer der Pandemie nun zentral.

Mit Blick auf die erste Fragestellung zu den Häufigkeiten psychischer Auffälligkeiten zeigten sich schulformbezogene Unterschiede. Gerade bei jüngeren Kindern im Grundschulalter sind psychische Auffälligkeiten deutlich angestiegen. Dies zeigte sich vor allem bei Verhaltensauffälligkeiten sowie Unaufmerksamkeit/Hyperaktivität. Schuleingangsuntersuchungen geben zudem Hinweise darauf, dass Kinder infolge der pandemiebedingten Einschränkungen bereits vor dem Schulbeginn Defizite in der sprachlichen Entwicklung zeigen, aber auch mit Verhaltensauffälligkeiten und psychosomatischen Beschwerden belastet sind [28]. Es ist damit zu rechnen, dass gerade in Grundschulen die psychischen und psychosomatischen Belastungen der SchülerInnen fortbestehen und ein erhöhter Bedarf an präventiven Maßnahmen besteht. Hierbei können Maßnahmen, wie beispielsweise gemeinsame gruppenstiftende Bewegungs- und Entspannungsaktivitäten (z. B. durch Ausflüge, Sportveranstaltungen, Bewegungs‑/Yogaangebote in der Hortzeit), hilfreich sein.

Auch bei den weiterführenden Schulen zeigte sich mit dem Pandemiebeginn ein deutlicher Anstieg psychischer Auffälligkeiten, insbesondere bei Verhaltensproblemen mit Gleichaltrigen. SchülerInnen der Haupt‑, Real- und Gesamtschulen zeigten insgesamt häufiger psychische Auffälligkeiten im Vergleich zu Gleichaltrigen an Gymnasien oder Fachoberschulen. Dies deckt sich mit Ergebnissen einer Studie aus Nordrhein-Westfalen, die ebenfalls signifikante Unterschiede zwischen den weiterführenden Schulen, insbesondere ein geringeres psychisches Wohlbefinden bei der Gruppe der Haupt‑/Sekundar‑, Real- und Gesamtschule, im Vergleich zum Gymnasium zeigte [6]. Heranwachsende aus Familien mit einem hohen Sozialstatus besuchen häufiger das Gymnasium [29] und gerade in stressvollen Lebenssituationen entwickeln Kinder und Jugendliche mit einem hohen Sozialstatus weniger psychische Auffälligkeiten [30] und zeigen weniger Bildungsrückstände [31, 32]. Bei den Förder- und Sonderschulen zeigten sich mit Beginn der COVID-19-Pandemie teils stark divergente Verläufe im Vergleich zu den anderen Schulformen, beispielsweise in Bezug auf Probleme mit Gleichaltrigen und Unaufmerksamkeit/Hyperaktivität. Weiterführende Forschungsarbeiten zu den unterschiedlichen Dynamiken der psychischen Gesundheit von Förder- und SonderschülerInnen sind empfehlenswert.

Mit Blick auf die zweite Fragestellung verdeutlichen die Ergebnisse der COPSY-Studie, dass sich seit Befragungsbeginn im Frühjahr 2020 mehr als drei Viertel der Eltern durch die Veränderungen der COVID-19-Pandemie belastet fühlten.

Die COPSY-Studie liefert somit erstmals repräsentative Daten über die psychische Gesundheit von Kindern und Jugendlichen und familiäre Belastungen im Verlauf der COVID-19-Pandemie. Eine Limitation ist, dass die COPSY-Befragung eine bevölkerungsbezogene Langzeitstudie und keine Schulerhebung ist. Sie richtet sich primär an Kinder und Jugendliche im Alter von 7 bis 17 Jahren, sodass ältere SchülerInnen (Klassenstufe 12 und 13) tendenziell unterrepräsentiert sind. Zudem können schulformspezifische Unterschiede bei Prävalenzen psychischer Auffälligkeiten in der Pandemie nicht nur auf Unterschiede der Schulform, sondern womöglich auch auf das Alter sowie sozioökonomische Unterschiede und einen Migrationshintergrund zurückzuführen sein. Hier bedarf es komplexerer Modelle mit Adjustierungen, um mögliche Zusammenhänge aufzeigen zu können.

Mit Blick auf die dritte Fragestellung gaben im Mittel etwa 70 % der Eltern von Grund- und Sonder‑/FörderschülerInnen sowie 61 % der Eltern von Haupt‑, Real- und GesamtschülerInnen einen Unterstützungsbedarf an. Dabei wünschten sich die Eltern vor allem Unterstützung durch die Schule und LehrerInnen sowie durch persönliche Gespräche und Beratung von ExpertInnen. In der repräsentativen Trendstudie *Jugend in Deutschland *aus dem Sommer 2022 gaben die Befragten an, sich mehr Unterstützungsangebote im schulischen Raum zu wünschen, vor allem mehr Information und Aufklärung, weniger Stigmatisierung und einen leichteren Zugang zu Hilfsangeboten [33]. Der Bedarf nach einer Anbindung an regionale ExpertInnen kann in einem ganzheitlichen Ansatz wie dem der gesundheitsfördernden Schule durch die Einbeziehung außerschulischer Akteure gut gedeckt werden. In der Umsetzung wurde international und auch bundesweit eine Vielzahl von Programmen zur Förderung der psychischen Gesundheit von Kindern und Jugendlichen im Schulalter entwickelt [34, 35]. Eine gelungene Gesundheitsförderung kann dabei als das Zusammenspiel von gesundheitsförderlichen Lebensverhältnissen und verhaltensbezogenen Maßnahmen verstanden werden. Thematische Schwerpunkte entsprechender Interventionen umfassen beispielsweise die emotionale Entwicklung sowie den Umgang mit Stress und Mobbing, wobei der Gesundheitskompetenz eine zunehmende Bedeutung beigemessen wird [16]. Mit der COVID-19-Pandemie zeigen sich auch vor dem Hintergrund der Erkenntnisse der COPSY-Studie neue Themenschwerpunkte der Gesundheitsförderung und Prävention, wie z. B. der Umgang mit dem Gefühl von Einsamkeit und sozialer Isolation [36]. Auch die Stärkung sozialer Kompetenzen im Umgang mit Gleichaltrigen und die Förderung des Gemeinschaftsgefühls sowie die Förderung des gemeinsamen (Nach‑)Verarbeitens der Pandemie können hierbei von Bedeutung sein.

Bisher bestehende Maßnahmen weisen eine unterschiedliche Komplexität auf. Sie reichen von monothematischen Programmen mit begrenzter Intensität und Dauer [17] bis hin zu ganzheitlichen Maßnahmen, die auf eine Aktivierung schulischer und außerschulischer Personengruppen und die Entwicklung und Umsetzung eigener Maßnahmen abzielen. Während Erstere häufig einen höheren Grad an Standardisierung aufweisen und vergleichsweise gut evaluiert sind [37], haben ganzheitliche Maßnahmen eine höhere Wahrscheinlichkeit der nachhaltigen Verankerung, werden aber aufgrund ihrer Komplexität deutlich seltener evaluiert.

Exemplarisch für eher verhaltensorientiert ausgerichtete Programme ist das international anerkannte Programm „Zippy’s Friends“, welches im Vorschulalter und im Grundschulalter über 24 Sitzungen à 45 min im Klassenraum umgesetzt wird [38]. Evaluationsbefunde weisen auf eine teils geschlechtsspezifische Verbesserung positiver Copingstrategien, eine Verbesserung sozialer Fähigkeiten und eine Reduktion psychischer Probleme hin [39]. „MindMatters“ ist ein ganzheitliches Programm mit australischen Wurzeln, das von 2002 bis 2005 adaptiert und seither in Deutschland weiterentwickelt und umgesetzt wird [40, 41]. Neben Unterrichtsmodulen für die Sekundarstufe 1 (z. B. mit Stress umgehen, Freunde finden) und einem Modul für die Primarstufe (gemeinsam(es) Lernen mit Gefühl) umfasst MindMatters Module, mit denen Schulen bei der gesundheitsförderlichen Organisationsentwicklung unterstützt werden. Bisherige Evaluationsbefunde weisen einen geringen Evidenzgrad auf, weshalb aktuell für das Grundschulmodul eine multizentrische cluster-randomisierte Kontrollgruppenstudie umgesetzt wird. Schließlich lässt sich das Projekt „Verrückt? Na und!“ anführen, das neben Fortbildungen für Lehrkräfte den persönlichen Kontakt zwischen SchülerInnen und Menschen mit psychischen Problemen/Erkrankungen herstellt. Evaluationsbefunde weisen auf eine hohe Akzeptanz der Schulcoaches, eine kurzfristige Abnahme der sozialen Distanz zu Menschen mit psychischen Erkrankungen und eine höhere Bereitschaft, mit Lehrkräften über psychische Probleme zu sprechen, hin [42, 43].

Verlässliche Daten über Art, Dauer und Umfang der Maßnahmen an Schulen gibt es derzeit nicht. Zwar ergab eine Befragung von Schulleitungen, dass 42 % der befragten Schulen einen nicht näher spezifizierten Schwerpunkt Gesundheit haben [44], jedoch erschweren die Auswirkungen der COVID-19-Pandemie für Schulen auch die Umsetzung schulischer Gesundheitsförderung [45].

## Fazit und Empfehlungen

Auch nach mehr als 2 Jahren Pandemie zeichnet sich kein ausreichender Rückgang psychischer Auffälligkeiten bei Kindern und Jugendlichen in Deutschland ab. Neue gesellschaftliche Krisen kommen hinzu und können Sorgen und Ängste bei Familien hervorrufen. Die Ergebnisse der COPSY-Studie zeigen, dass vor allem in Grundschulen sowie Haupt‑/Real- und Gesamtschulen psychische Auffälligkeiten mit dem Beginn der COVID-19-Pandemie deutlich angestiegen sind. Handlungsbedarf formulieren Wissenschaftsgremien, wie die Leopoldina [46] und der Deutsche Ethikrat [47], z. B. in Hinblick auf die Fortbildung pädagogischer Fachkräfte in Schulen im Sinne eines Frühwarnsystems, den Ausbau der Schulsozialarbeit und psychosozialer Beratungsangebote, die Förderung eines gesunden Lebensstils in Schulen sowie spezifische Konzepte für sozial benachteiligte und vulnerable Gruppen von Kindern und Jugendlichen.

Maßnahmen der schulischen Gesundheitsförderung und Prävention werden auch zukünftig eine hohe Relevanz in der Public-Health-Forschung haben. Dennoch gibt es im Setting Schule keine bundesweit verlässlichen Daten über die Art und den Umfang der Maßnahmen, die an Schulen angeboten werden. Es besteht ein dringender Bedarf an einem bundesweiten kontinuierlichen Gesundheitsmonitoring von Kindern und Jugendlichen im Kontext Schule, wobei auf bereits existente Strukturen zurückgegriffen werden sollte (beispielsweise die HBSC-Studie). Bundesländerübergreifende Daten sind notwendig, um (1) Trends zur physischen und psychischen Gesundheit und dem Gesundheitsverhalten von Kindern und Jugendlichen zu erhalten, (2) Bedarfe zu ermitteln (schulspezifisch, themenspezifisch) und (3) eine Datenbasis zur Evaluation von Maßnahmen der Gesundheitsförderung und Prävention zu etablieren. Ganzheitliche Konzepte im Sinne eines Whole School Approach versprechen eine größere Nachhaltigkeit, müssen aber künftig mittels evidenzstarker Studiendesigns besser evaluiert werden. Vor allem in Grundschulen sowie Haupt‑/Real- und Gesamtschulen ist es dringend notwendig, Maßnahmen zur Förderung der mentalen Gesundheit und Prävention psychischer Erkrankungen zu fördern. Da Schule per se niedrigschwellig ansetzt, ist sie auch geeignet, Kinder aus sozial benachteiligten Familien zu erreichen (statt Präventionsdilemma^1^). Eine bundesweit verbindliche gesetzliche Verankerung von Gesundheitsförderung und Prävention an Schulen kann helfen, geeignete gesundheitsfördernde Strukturen aufzubauen sowie zeitliche, personelle und finanzielle Ressourcen für die Umsetzung und nachhaltige Implementierung zu schaffen.

## Supplementary Information





## Footnotes

1 Der Begriff Präventionsdilemma beschreibt die Schwierigkeit, besonders vulnerable Personengruppen für die Teilnahme an Maßnahmen der Gesundheitsförderung und Prävention zu erreichen, obwohl bei diesen ein besonders hoher Präventionsbedarf besteht.
