# Supplementary material for: Prevention perspective: mental health of schoolchildren in Germany
Source: Bundesgesundheitsblatt Gesundheitsforschung Gesundheitsschutz. 2023 Mar 10;66(4):391–401. [Article in German] doi: 10.1007/s00103-023-03674-8 (PMC10005858; doi:10.1007/s00103-023-03674-8)

Perspektive Prävention: Psychische Gesundheit von Schülerinnen und Schülern in Deutschland

AutorInnen: Franziska Reiß[1], Ann-Kathrin Napp[1], Michael Erhart[1,2,3], Janine Devine[1], Kevin Dadaczynski[4], Anne Kaman[1], Ulrike Ravens-Sieberer[1]

Instituts- und Klinikangaben

[1] Universitätsklinikum Hamburg-Eppendorf, Zentrum für Psychosoziale Medizin, Klinik für Kinder- und Jugendpsychiatrie, -psychotherapie und -psychosomatik, Hamburg, Deutschland

[2] Alice Salomon Hochschule, Berlin, Deutschland

[3] Apollon Hochschule der Gesundheitswirtschaft, Bremen, Deutschland

[4] Hochschule Fulda, Fachbereich Gesundheitswissenschaften, Fulda, Hessen, Deutschland

Korrespondenzadresse

Prof. Dr. Ulrike Ravens-Sieberer

Universitätsklinikum Hamburg-Eppendorf

Zentrum für Psychosoziale Medizin, Klinik für Kinder- und Jugendpsychiatrie,

-psychotherapie und -psychosomatik, Forschungssektion Child Public Health

Martinistraße 52, 20246 Hamburg

E-Mail: ravens-sieberer@uke.de

Telefon: 040 7410-52992

Zusätzliches Onlinematerial

Abbildung Z1. Subskalen psychischer Auffälligkeiten vor der COVID-19-Pandemie und im Verlauf der COVID-19-Pandemie nach Schulform (Daten gewichtet)

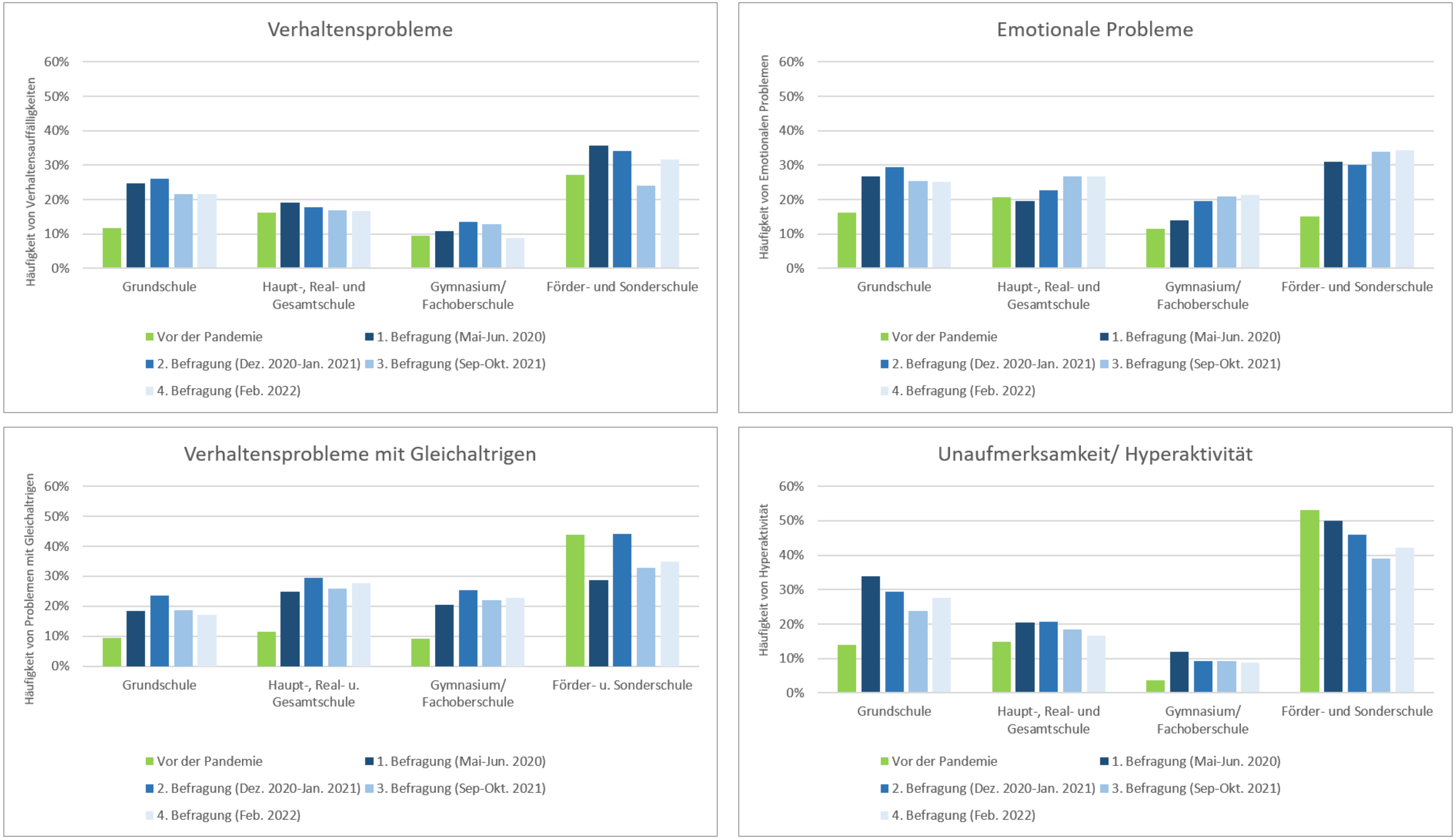

Supplement: Supplementary file 1 [file 103_2023_3674_MOESM1_ESM.pdf]
